# Polyploidy of Endosymbiotically Derived Genomes in Complex Algae

**DOI:** 10.1093/gbe/evu071

**Published:** 2014-04-07

**Authors:** Yoshihisa Hirakawa, Ken-Ichiro Ishida

**Affiliations:** Faculty of Life and Environmental Sciences, University of Tsukuba, Ibaraki, Japan

**Keywords:** chlorarachniophyte, cryptophyte, endosymbiosis, nucleomorph, plastid

## Abstract

Chlorarachniophyte and cryptophyte algae have complex plastids that were acquired by the uptake of a green or red algal endosymbiont via secondary endosymbiosis. The plastid is surrounded by four membranes, and a relict nucleus, called the nucleomorph, remains in the periplastidal compartment that is the remnant cytoplasm of the endosymbiont. Thus, these two algae possess four different genomes in a cell: Nuclear, nucleomorph, plastid, and mitochondrial. Recently, sequencing of the nuclear genomes of the chlorarachniophyte *Bigelowiella natans* and the cryptophyte *Guillardia theta* has been completed, and all four genomes have been made available. However, the copy number of each genome has never been investigated. It is important to know the actual DNA content of each genome, especially the highly reduced nucleomorph genome, for studies on genome evolution. In this study, we calculated genomic copy numbers in *B. natans* and *G. theta* using a real-time quantitative polymerase chain reaction approach. The nuclear genomes were haploid in both species, whereas the nucleomorph genomes were estimated to be diploid and tetraploid, respectively. Mitochondria and plastids contained a large copy number of genomic DNA in each cell. In the secondary endosymbioses of chlorarachniophytes and cryptophytes, the endosymbiont nuclear genomes were highly reduced in size and in the number of coding genes, whereas the chromosomal copy number was increased, as in bacterial endosymbiont genomes. This suggests that polyploidization is a general characteristic of highly reduced genomes in broad prokaryotic and eukaryotic endosymbionts.

## Introduction

Plastids (chloroplasts) have been acquired through multiple endosymbiotic events. Land plants and three algal groups (green, red, and glaucophyte algae) acquired plastids via a primary endosymbiosis of a photosynthetic cyanobacterium (Rodríguez-Ezpeleta et al. 2005; Price et al. 2012). In contrast, other algae have more complex plastids, called secondary plastids, which originated from the ingestion of primary plastid-bearing algae such as green and red algae (Archibald 2009; Keeling 2010). Secondary plastids are thus bound by one or two additional membranes in comparison with primary plastids which are bound by two membranes. Chlorarachniophytes and cryptophytes have secondary plastids derived from a green and red algal endosymbiont, respectively (Curtis et al. 2012). These two algal groups are of special interest, because the relict nucleus, called the nucleomorph, of engulfed algae still exists in the periplastidal compartment (PPC), which is the space between the inner and outer pair of plastid membranes, whereas many other algae have lost this organelle (Archibald 2007; Moore and Archibald 2009).

Nucleomorphs contain a greatly reduced genome that is the smallest known eukaryotic nuclear genome. Complete nucleomorph genomic sequences have been reported in a chlorarachniophyte (Gilson et al. 2006) and four cryptophyte species (Douglas et al. 2001; Lane et al. 2007; Tanifuji et al. 2011; Moore et al. 2012). The nucleomorph genomes sequenced to date consist of three linear chromosomes with ribosomal DNA operons on all six chromosome ends. The genomes are extremely small and compact ranging in size from 373 to 703 kb with only several hundred genes. The structural similarities of nucleomorph genomes between chlorarachniophytes and cryptophytes are striking when one considers that they are of independent origin. All nucleomorph genomes encode a small number of plastid proteins and hundreds of housekeeping proteins, suggesting that nucleomorphs are essential to maintain plastids. However, numerous fundamental proteins for nucleomorph biogenesis are absent from nucleomorph genomes (e.g., DNA polymerase genes). Recently, complete nuclear genomes have been sequenced in the chlorarachniophyte *Bigelowiella natans* and the cryptophyte *Guillardia theta* (Curtis et al. 2012). These revealed that thousand(s) of nucleus-encoded proteins are imported into the PPC to compensate for the lacking proteins. In secondary endosymbioses, numerous genes were lost and transferred from the endosymbiont nuclei to the host nuclei, and consequently the highly reduced nucleomorph genomes were generated.

Two major organelles, chloroplasts and mitochondria, were derived from bacterial endosymbionts, and they generally contain a large copy number of genomic DNA (plastid DNA [ptDNA] and mitochondrial DNA [mtDNA]). For instance, the copy numbers of ptDNA and mtDNA in the unicellular red alga *Cyanidioschyzon merolae* have been estimated to be 24 and 9 per organelle, respectively (Moriyama et al. 2010), and approximately 80 ptDNA and several hundred mtDNA copies have been predicted to exist in a single cell of the green alga *Chlamydomonas reinhardtii* (Nishimura et al. 1998). In contrast, many bacterial species generally possess a single copy or a small number of chromosome(s); the model proteobacterium, *Escherichia coli*, contains a single copy of the chromosome (Lau et al. 2003), and the cyanobacterium, *Synechococcus elongatus*, is known to possess one to eight copies of the circular chromosome (Chen et al. 2012). These imply that bacterial endosymbiont genomes tend to increase their copy numbers to be polyploid organelle genomes. This feature has also been seen in the bacterial endosymbiont of insects, *Buchnera*, which has more than 100 copies of a highly reduced genome (Komaki and Ishikawa 1999). However, reports of polyploidization in endosymbiotically derived genomes have been limited to prokaryotic genomes. To investigate the generality of polyploidization in broad endosymbiotic genomes, we analyzed the ploidy of endosymbiotically derived nuclear genomes in two algal groups, chlorarachniophytes and cryptophytes.

In this study, we calculated copy numbers of the four genomes in the chlorarachniophyte, *B. natans*, and the cryptophytes, *G. theta*, using a real-time quantitative polymerase chain reaction (qPCR) approach. These species have a single nucleus, nucleomorph, plastid, and mitochondrion in a cell, which is suitable for estimating genomic copy numbers (Hill and Wetherbee 1990; Moestrup and Sengco 2001). Our data indicate that the nuclear genomes are haploid, whereas the nucleomorphs are diploid or tetraploid. The mitochondrion and plastid contained a large copy number (20 to over 200 copies) of genomic DNA. We also analyzed relative transcript levels of homologous ribosomal protein genes in the four genomes of *B. natans* and the three genomes of *G. theta* by reverse transcription qPCR. The mRNA levels were increased in the nuclear, nucleomorph, mitochondrial, and plastid genes in the order of representation, which correlated with genomic copy numbers. Together, our data suggest that endosymbiotically derived genomes tend to be polyploid even in the case of eukaryotic nuclear genomes and that gene transcription appears to be increased according to the degree of polyploidy.

## Materials and Methods

### DNA and RNA Extraction from Cell Culture

*Bigelowiella natans* (CCMP621) and *G. theta* (CCMP2712) cells were grown at 20 °C under white illumination on a 12 h/12 h light/dark cycle in ESM medium (Kasai et al. 2009). Fresh cultures in midlight phase (predicted G1-phase) were used for DNA and RNA extractions, as most nuclear DNA replication and cell division of *B. natans* are performed in the dark phase (Hirakawa et al. 2011). DNA was purified using a DNeasy Plant Mini Kit (Qiagen) and a Plant DNA Preparation Kit (Jena Bioscience) for real-time qPCR. To quantify the amount of DNA per cell, total DNA was extracted from cells by a high-yield purification method using the Plant DNA Preparation Kit (without phenol/chloroform and spin columns). Cultured cell density (cells/ml) was calculated using a hemocytometer. DNA amount was measured using a microvolume spectrophotometer (NanoDrop 1000, Thermo Scientific). Total RNA was extracted using Trizol Reagent (Invitrogen) according to the manufacturer’s protocol. cDNA was synthesized from 3 µg total RNA using a ReverTra Ace qPCR RT Kit (Toyobo) with random 9-mer primers in a total reaction volume of 40 µl.

### Real-Time Quantitative PCR

PCR primers for single-copy genes from each genome were designed using Primer3Plus software (http://www.bioinformatics.nl/cgi-bin/primer3plus/primer3plus.cgi/primer3plus.cgi, last accessed April 17, 2014), based on the sequences of nuclear genomes (Curtis et al. 2012), nucleomorph genomes (Douglas et al. 2001; Gilson et al. 2006), plastid genomes (Douglas and Penny 1999; Rogers et al. 2007), and mitochondrial genomes (GenBank: HQ840955; GQ896379) for *B. natans* and *G. theta*; target genes and primer sequences are shown in table 1 and supplementary table S1, Supplementary Material online. Each fragment was amplified and cloned into a pGEM-T easy vector (Promega), and serial dilutions of the plasmids were used to create standard curves. Real-time qPCR was carried out using a Thermal Cycler Dice Real Time System II (Takara) under the following conditions: 10 ng of DNA/0.5 µl of cDNA, 0.4 µM of each primer, 12.5 µl of SYBR Premix Ex Taq II (Takara), and DNase/RNase-free water up to 25 µl. The cycling conditions were as follows: 3 min of denaturation at 95 °C followed by 40 cycles of 10 s at 95 °C and 30 s at 60 °C, and a melting curve program for detection of nonspecific products. The absolute copy number of target DNA/cDNA were calculated by the Ct values (2nd derivative maximum) and the standard curves of serial dilutions. The qPCR analysis was repeated at least three times for estimating standard deviations.

## Results

### Copy Numbers of the Four Genomes in a Chlorarachniophyte and a Cryptophyte

To estimate copy numbers of the four genomes (nuclear, nucleomorph, mitochondrial, and plastid) in the chlorarachniophyte *B. natans* and the cryptophyte *G. theta*, we used an absolute quantification method by real-time qPCR with genome-specific primers. The primers were designed on single copy genes of each genome; two primer sets were designed specific to the nuclear/plastid/mitochondrial genomes except for a single set in the *G. theta* mitochondrial genome, and three primer sets were designed for the three nucleomorph chromosomes (table 1). All target fragments were amplified by PCR, cloned into plasmid vectors, and serial dilutions of the plasmids were used to create standard curves for calculating exact DNA copy numbers. Total DNA was extracted from different batches of cell cultures in three independent experiments (samples A–C) and used for qPCR analyses.

**Table 1:** Primer Sequences for Quantitative PCR

Target Genome	Coding Gene	Forward Primer	Reverse Primer
BnN #1	rbcS2	TCGCTAACCTGCTGACTCTTTG	TTGACATTGCTGCTCTGGTTG
BnN #2	atpD	ACTTCAAACTTCTTGAACCTTCTCC	CATCTTCATCACTTCTTCCTCAAAC
BnNm #1	rpL8 (Ch 1)	TACTTACCGCCACCTGCTACAAC	GCCTTTGTCGGTGTTCTTTCTC
BnNm #2	cdc48 (Ch 2)	TTCTCCTACGCCCACATACTTACTC	TGGCATTTTACTTTACGGACCAC
BnNm #3	eef2 (Ch 3)	GGAGGGGAAAAGTGTATGAGGAG	AATGCCTGCCCTGATGTAGAAG
BnM #1	nad9	TGGGATTTGCTTGGTGTGTTC	GTCTTTTCGTAGTGGGTGTCCTTC
BnM #2	nad3	AGAAAAGCTATCCGCCTATGAGTG	AAACGGCTCAAGGAAATAGAAGG
BnP #1	psbD	CAAGATCAACCGCACGAGAAG	GAGCATTTCCAGACCACCAAG
BnP #2	petA	TCAAGAAGGGCAACAGGTAAAAG	CAGTTTCAGATTGACCAAATCCAC
GtN #1	rpL8	GTCGTGGTGCTGGTTCTGTC	CCATTCCTCTCAGCAAAGTCC
GtN #2	rpL15	CAACGATCCTCGCCTCAAC	ACCTGTGATTGCCGTGTCC
GtNm #1	rpb2 (Ch 1)	TTGCCTTTAGCGGATGGTG	CGGAAACTGGGGAACAGATAAG
GtNm #2	gidA (Ch 2)	AGCCATTTCTCCTGACCTTCC	GGATTTTACCGATGCCGTTC
GtNm #3	kin (Ch 3)	GAAGCGGATCATTTGGAGTTG	CTCTGTTTTCATACCTGTCGTCTTG
GtM #1	cox1	GTCAACCCTTGGGCATTTTC	AACGTGTGAAATGATGGTGGAG
GtP #1	atpH	TCTTCCTTCTGCTTCTGGTTGG	CTGCTTCTGTTGTTGCTTCTGG
GtP #2	rpoB	TTAATGCGTGCGTGTGTTG	CGTGATAGAGCAGGTTTTGGTG

Note.—Bn, *Bigelowiella natans*; Gt, *Guillardia theta*; N, nucleus; Nm, nucleomorph; M, mitochondrion; P, plastid; Ch, chromosome.

**Fig. 1.—:**
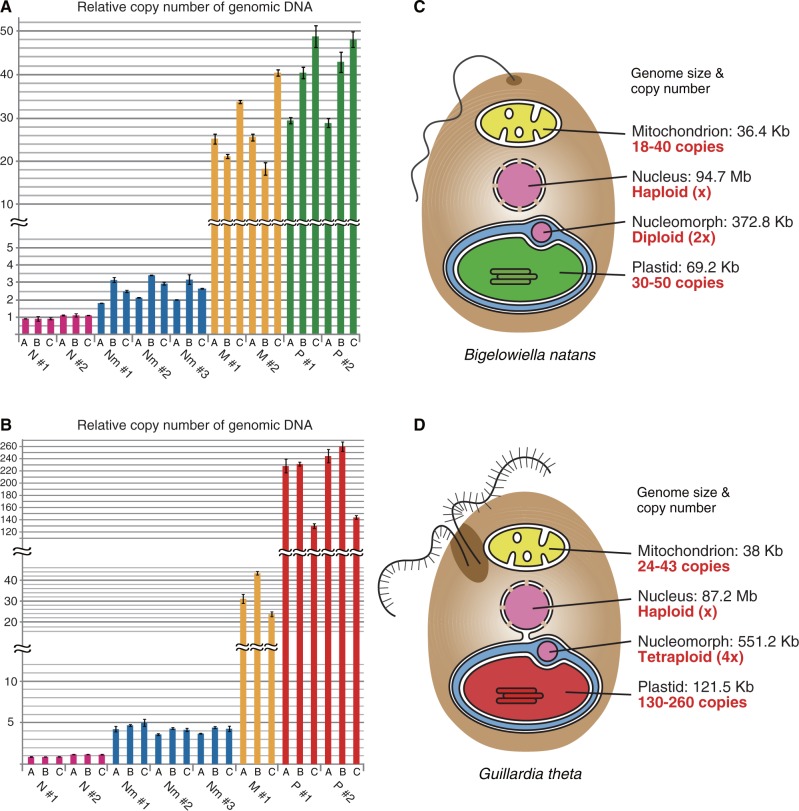
Copy numbers of the four genomes in the chlorarachniophyte, *Bigelowiella natans*, and the cryptophyte, *Guillardia theta*. (*A*, *B*) Each bar shows the relative copy numbers of genomes (N, nuclear; Nm, nucleomorph; M, mitochondrial; P, plastid) calculated by real-time quantitative PCR in three independent experiments (samples A–C). The average value of the nuclear genome was set to 1.0, and other values were normalized. Error bars represent the standard deviation (SD) (*n* = 3–4). (*C*, *D*) Size and copy number of each genome are summarized in the cellular schemes.

The copy numbers of nucleomorph chromosomes in *B. natans* and *G. theta* were predicted to be double to triple and quadruple that of nuclear chromosomes, respectively (fig. 1*A* and *B*). A stable copy number of nucleomorph chromosomes in *G. theta* was seen in three different DNA samples (fig. 1*B*), whereas the nucleomorph chromosome copy number of *B. natans* varied (fig. 1*A*). This variability was due to contamination by cells with divided plastids and nucleomorphs in the cultures; double plastids were detected by confocal laser microscopy in approximately 50% of cells (39 out of 75 cells) in sample C that showed a triple copy number of nucleomorph chromosomes (fig. 1*A*, supplementary fig. S1, Supplementary Material online). This suggests that the chromosome copy number of a single nucleomorph in *B. natans* is double that of nucleus. Three nucleomorph chromosomes indicated the same copy number in both species (fig. 1*A* and *B*), as previously seen by Southern blot analyses of pulsed-field gel separation that showed identical signal intensities of the three nucleomorph chromosomes (McFadden et al. 1994; Lane and Archibald 2006). Copy numbers of the *B. natans* mitochondrial and plastid genomes were approximately 18–40 and 30–50 times greater than that of the nuclear genome, respectively (fig. 1*A*). For *G. theta*, the mitochondrion and plastid contained approximately 24- to 43-fold and 130- to 260-fold more genomic copies than the nucleus, respectively (fig. 1*B*).

Ploidy of the nuclear genomes of *B. natans* and *G. theta* was calculated from the amount of DNA per cell. Total DNA was extracted from cell cultures by a high-yield solution-based purification method without using phenol/chloroform and spin columns, and its weight was quantified by the absorbance at 280 nm (NanoDrop 1000). It was estimated that a single cell contained from 8.6 × 10^−^^2^ to 9.1 × 10^−^^2 ^pg DNA in *B. natans*, and from 10.0 × 10^−^^2^ to 11.4 × 10^−^^2 ^pg DNA in *G. theta*, based on experiments performed in triplicate. Assuming that the nuclear genomes are haploid, the total DNA content was calculated to be 10.4 × 10^−^^2^ and 11.8 × 10^−^^2 ^pg/cell for *B. natans* and *G. theta*, respectively, based on the estimated total nucleotides of four genomes (genome size multiplied by the copy number) and a factor used to convert DNA weight to the number of base pairs (0.978 × 10^−^^9^) (Dolezel et al. 2003), using the following formulas: (94.7 × 10^6 ^× 1 + 372.8 × 10^3 ^× 2 + 69.2 × 10^3 ^× 40 + 36.4 × 10^3 ^× 27)/0.978 × 10^−^^9 ^= 10.4 × 10^−^^2^ (pg/cell) for *B. natans*, and (87.2 × 10^6 ^× 1 + 551.2 × 10^3 ^× 4 + 121.5 × 10^3 ^× 206 + 38 × 10^3 ^× 33)/0.978 × 10^−^^9 ^= 11.8 × 10^−^^2^ (pg/cell) for *G. theta*. The above data indicate that the nuclear genomes consist of single copy chromosomes in both species. Altogether, these results suggest that the nucleomorph genomes are diploid (2 *n* = 6) in *B. natans* and tetraploid (4 *n* = 12) in *G. theta* and that the plastids and mitochondria contain a large copy number of genomic DNA.

### Transcript Levels of Homologous Genes in the Four Genomes

**Fig. 2.—:**
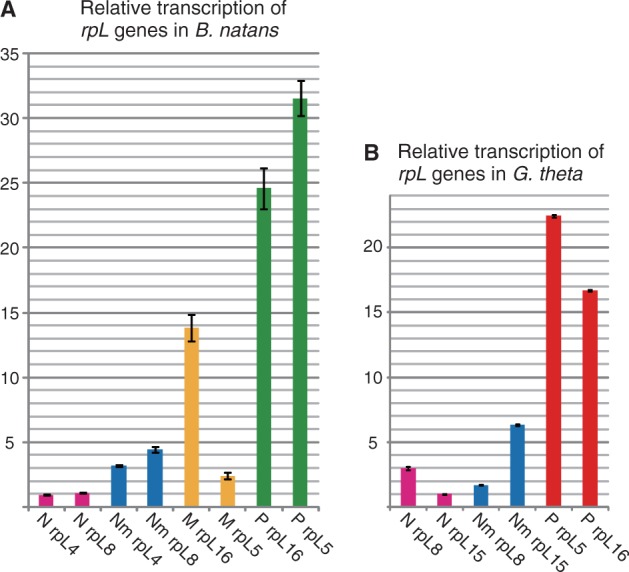
Relative transcription levels of homologous genes residing in the four genomes of *Bigelowiella natans* and the three genomes of *Guillardia theta*. (*A*, *B*) Each bar shows the relative transcript levels of ribosomal protein genes calculated by real-time quantitative PCR (N, nuclear; Nm, nucleomorph; M, mitochondrial; P, plastid). The value of nuclear *rpL8* genes was set to 1.0, and other values were normalized. Error bars represent the standard deviation (SD) (*n* = 3).

Both *B. natans* and *G. theta* possess multiple copies of organelle genomes (nucleomorph, mitochondrial, and plastid), and these genomes show different copy numbers. To determine the relationship between genomic copy number and mRNA expression in each genome, we examined transcription of homologous genes in each genome of *B. natans* and *G. theta*. Several ribosomal protein genes, *rpL4* and *rpL8* of *B. natans* and *rpL8* and *rpL15* of *G. theta*, were used to calculate the relative transcript levels of nuclear and nucleomorph genes, and *rpL5* and *rpL16* were used for plastid and/or mitochondrial genes of both species. cDNA was synthesized from total RNA using random primers (9 mers), and the relative transcription of each gene was quantified by real-time qPCR with gene-specific primers (supplementary table S1, Supplementary Material online). Transcript levels of the nucleomorph *rpL* genes were 3–4-fold higher than those of their nuclear counterparts with the exception of *G. theta rpL8*. (fig. 2*A* and *B*). Although we cannot compare the transcription of exact homologous genes between eukaryotic and prokaryotic type genomes, transcript levels of the plastid and/or mitochondrial *rpL* genes were generally greater than those of the nuclear and nucleomorph *rpL* genes in both *B. natans* and *G. theta* (fig. 2*A* and *B*). Transcript levels of *rpL* genes largely increased from the nuclear, nucleomorph, mitochondrial, to plastid genomes in that order, which seems to be positively correlated with the copy number of each genome.

## Discussion

In this study, we show that the nucleomorphs of the chlorarachniophyte *B. natans* and the cryptophyte *G. theta* contained a diploid and tetraploid genome, respectively, whereas their nuclear genomes were haploid. The nucleomorphs are a relict nucleus of a green or red algal endosymbiont (Douglas et al. 2001; Gilson et al. 2006). Although in algae the chromosome copy numbers can vary at different stages of the life cycles, the genomes of Chlorophyta and Rhodophyta gametophytes have been typically reported as haploid (Kapraun 2007; Kapraun and Freshwater 2012). Thus, copy numbers of chromosomes in nucleomorph genomes seem to have multiplied after the endosymbiotic events. Interestingly, the nucleomorph genomes of *B. natans* and *G. theta* are highly reduced in size (373 and 551 kb) and coding genes (284 and 487 genes), whereas increased in their chromosomal copy numbers. This feature has been observed in other endosymbiotically derived genomes of plastids and mitochondria. Both organelles contain greatly reduced multiple copy genomes, though the relatives of their origins, proteobacteria and cyanobacteria, have a single or a few genomic copies. Furthermore, the bacterial symbiont of aphids, *Buchnera*, has more than a hundred copies of a highly reduced genome, despite a single genome copy in the closely related bacterium *E. coli* (Komaki and Ishikawa 1999). Therefore, we hypothesize that genomic polyploidization is a general characteristic in the highly reduced genomes of both prokaryotic and eukaryotic endosymbionts.

The evolutionary advantages of genomic polyploidy in flowering plants and animals have been discussed in several reviews (Comai 2005; Sémon and Wolfe 2007; Arrigo and Barker 2012); for example, gene expression changes, buffering of deleterious mutations, and sub- and neofunctionalization of duplicated genes. Although the functional significance of polyploidy for nucleomorph genomes is unclear, the increased gene dosage would allow for higher productivity in the PPC. In fact, transcript levels of homologous genes were higher in the diploid/tetraploid nucleomorph genomes than in the haploid nuclear genomes of *B. natans* and *G. theta* (fig. 2). Very recently, a high level of mRNA transcription for *B. natans* and *G. theta* nucleomorph genes was reported based on RNA-seq transcriptome data (Tanifuji et al. 2014). The authors hypothesized that the high gene expression compensates for an inefficiency of protein complexes functioning in the PPC, because part of the fundamental proteins were absent from the PPC (e.g., 17 of 79 ribosomal protein subunits are not present). Although there is no proof for this hypothesis, polyploidy of the nucleomorph genome appears to be related to the high expression of nucleomorph genes.

Nucleomorph genomes of both chlorarachniophytes and cryptophytes have a gene-dense structure with shrinking intergenic regions (Douglas et al. 2001; Gilson et al. 2006). Many syntenic regions with the same gene order among different species were found in cryptophyte nucleomorph genomes (Lane et al. 2007; Tanifuji et al. 2011; Moore et al. 2012), as well as draft genomes of chlorarachniophyte nucleomorphs. Overlapping gene transcription has also been reported in nucleomorph genomes (Williams et al. 2005). It is known that gene-dense genomes generally show a low rate of viable genetic rearrangements, because nonhomologous recombination is likely to disrupt coding sequences. Indeed, nucleomorph genomes are composed of many syntenic regions; however, many intra- and interchromosomal recombination of syntenic blocks have also been reported in nucleomorph genomes (Lane et al. 2007). This implies that the multiple copies of nucleomorph genomes would lead to an increase in the recombination frequency.

The replication and segregation systems of nucleomorph DNA are at present unknown, and chromosome structure has never been observed by electron microscopy. We previously reported that expression of nucleomorph histone genes was controlled during the cell cycle of the chlorarachniophyte *B. natans*; transcript levels of the histones increased in S-phase, which would correlate to nucleomorph chromosome replication (Hirakawa et al. 2011). This study indicates that nucleomorphs contain stable copy numbers of three chromosomes. Though there is no direct evidence, replication of nucleomorph chromosomes is likely tightly controlled by the cell cycle, and duplicated chromosomes are segregated to daughter plastids nonrandomly.

In this study, we investigated copy numbers of the four genomes in a chlorarachniophyte and cryptophyte and found that the nucleomorph genomes were diploid and tetraploid, respectively. It is interesting that two distantly related algal groups have individually evolved multiple copied genomes in endosymbiotically derived organelles. We hypothesize that genomic polyploidization may be a general characteristic of highly reduced genomes in endosymbionts. To test this hypothesis, genomic copy numbers should be investigated in other endosymbiotically derived genomes from a broad variety of organisms.

## Supplementary Material

Supplementary figure S1 and table S1 are available at *Genome Biology and Evolution* online.

Supplementary Data
