# Supplementary material for: Polyploidy of Endosymbiotically Derived Genomes in Complex Algae
Source: Genome Biol Evol. 2014 Apr 7;6(4):974–80. doi: 10.1093/gbe/evu071 (PMC4007541; doi:10.1093/gbe/evu071)
Supplement: Supplementary Data [file supp_6_4_974__index.html]

Polyploidy of endosymbiotically derived genomes in complex algae — Polyploidy of Endosymbiotically Derived Genomes in Complex Algae — Supplementary Data 

# Polyploidy of Endosymbiotically Derived Genomes in Complex Algae

## Supplementary Data

files

**Files in this Data Supplement:**

- Supplementary Data - pdf file
