# Supplementary material for: Polyploidy of Endosymbiotically Derived Genomes in Complex Algae
Source: Genome Biol Evol. 2014 Apr 7;6(4):974–80. doi: 10.1093/gbe/evu071 (PMC4007541; doi:10.1093/gbe/evu071)
Supplement: Supplementary Data [file supp_evu071_Supplemetal_data.pdf]

Figure S1

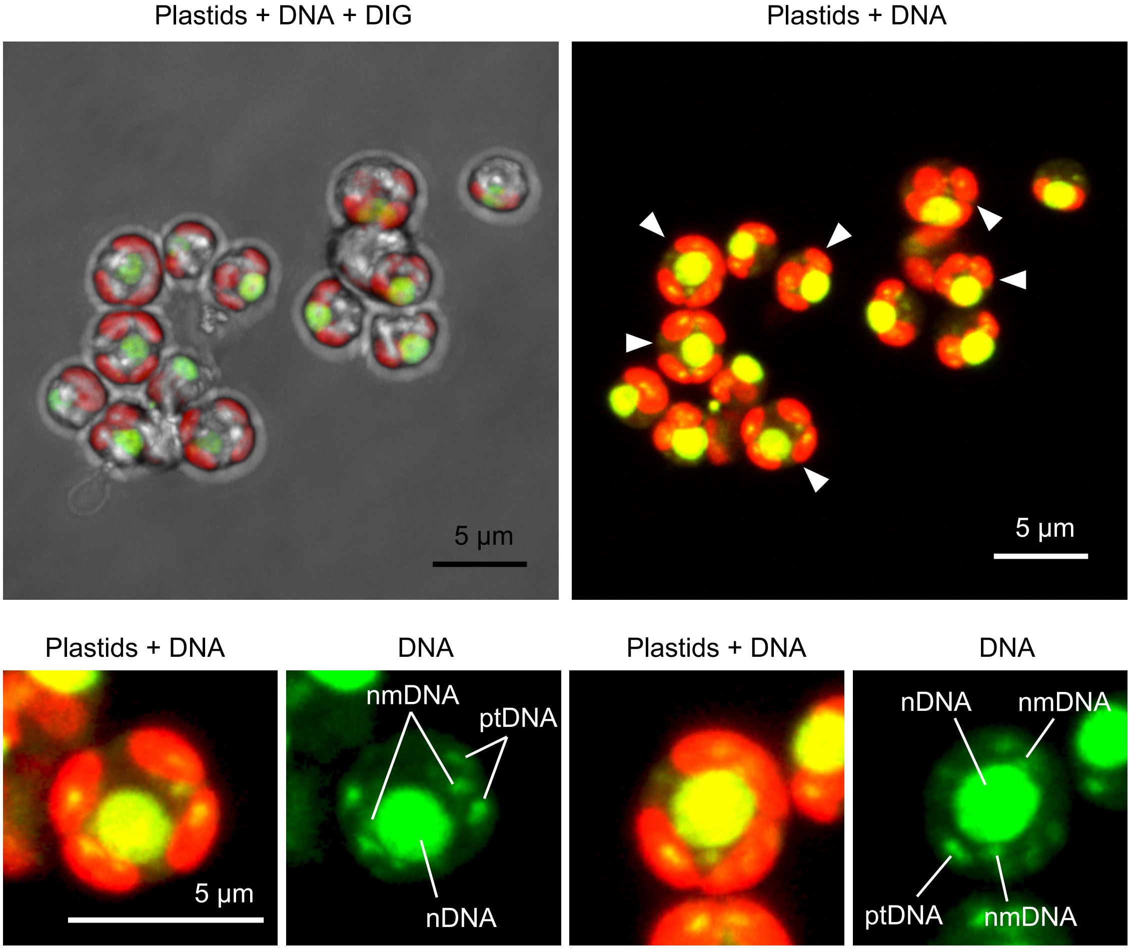

Figure S1. Three-dimensional images of plastid and DNA localization in *Bigelowiella natans* cells. The images were reconstructed from 17 scanning images obtained by a confocal laser microscope (Zeiss LSM 510). Red chlorophyll autofluorescence corresponds to plastids, and green/yellow fluorescence is showing DNA localization stained by SYBR Green. White allow heads indicate the cells possessing divided plastids.

nmDNA, nucleomorph DNA; ptDNA, plastid DNA; nDNA, nuclear DNA.

Table S1. Quantitative PCR primers for ribosomal protein genes of *Bigelowiella natans* and *Guillardia theta*

Organism	Target genome	Gene	Forward primer	Reverse primer
B. natans	**Nuclear**	rpL4	GAACATTCCTGGCGTTGAGG	CGCTTAGTGGAACCCCAGATAG
	**Nuclear**	rpL8	CCGCAACCCGTACAAATACAAG	GCATAGCCGAAAGAGGAAGAAC
	**Nucleomorph**	rpL4	ATAACTCTGGGAATCAAGTTTCAGC	TTTTCGTGTACCTGATCCCTTTATC
	**Nucleomorph**	rpL8	GCCTTTGTCGGTGTTCTTTCTC	TACTTACCGCCACCTGCTACAAC
	**Mitochondrial**	rpL5	AGGGCCTTTGATACAGGAATTTTAG	TCCAAGCGCATAGGCAAAC
	**Mitochondrial**	rpL16	TTAGTGGCCTAGAAGACAAAGCTG	GCTCATCGTAAACAAAACTGGTG
	**Plastid**	rpL5	ATTTAGCTCTGCCAAGAGTACGTG	GACCCAAACTAAAGTTACCACAACC
	**Plastid**	rpL16	ACCTATTACTTTCCGTCCACCAG	GATCTGACAACTGAAACCCAAAG
G. theta	**Nuclear**	rpL8	GTCGTGGTGCTGGTTCTGTC	CCATTCCTCTCAGCAAAGTCC
	**Nuclear**	rpL15	CAACGATCCTCGCCTCAAC	ACCTGTGATTGCCGTGTCC
	**Nucleomorph**	rpL8	TCATCAACATATCGGTCATCCTTC	GGTTCGTCTTGCTCCTATTAAACC
	**Nucleomorph**	rpL15	AAAACCCACTAGACCAGAGAAAGC	ATGACTCTTAGGCTTACCCGTTG
	**Plastid**	rpL5	AAATTAGAGAAGGTATGCCTGTTGG	CGAATACGCGGTAAAGAAAGATG
	**Plastid**	rpL16	TAAACGAGTTGGTCGATTGTGG	GCTCCCATTCTTGTTTCTGGTG
